# Staged treatment of osteonecrosis of the femoral head induced by delayed treatment of polytrauma complicated by chronic acetabular fracture and hip dislocation: a case report

**DOI:** 10.3389/fsurg.2026.1775612

**Published:** 2026-03-26

**Authors:** Deguo Luo, Shi Shen, Zhengrui Zhou, Benbiao Wang, Jianhua Ge, Naiqiang Zhuo

**Affiliations:** Department of Bone and Joint Surgery, Affiliated Hospital of Southwest Medical University, Luzhou, Sichuan, China

**Keywords:** acetabular fracture, damage control orthopedics, osteonecrosis of the femoral head (ONFH), polytrauma, staged treatment, total hip arthroplasty (THA)

## Abstract

Delayed intervention for hip fracture and dislocation is a core risk factor for osteonecrosis of the femoral head (ONFH). According to the latest research, the incidence of ONFH in patients with acetabular fracture combined with hip dislocation can reach 30%–40%, and this risk increases 1.89 times if treatment is delayed for more than 3 weeks. This presents a clinical challenge in polytrauma management, where systemic stability must be balanced against timely hip injury intervention under the principles of damage control orthopedics. This study reports the case of a 45-year-old man with severe polytrauma following a traffic accident. He suffered from extensive soft tissue injuries, bilateral pelvic and right acetabular comminuted fractures, central right hip dislocation, and multiple open injuries that required priority treatment. Repair of the right comminuted acetabular fracture with central hip dislocation was delayed for 53 days, ultimately resulting in secondary ONFH and requiring total hip arthroplasty (THA). Imaging examinations confirmed an Orthopaedic Trauma Association (OTA) acetabular fracture type 62-C, with a 2.5-cm displacement of the femoral head into the pelvic cavity. Staged interventions—including damage control, wound repair, hip reconstruction, and THA—were performed. At the 30-month follow-up, the patient demonstrated significant improvement in hip function [Harris Hip Score increased from 45 to 88 points, visual analog scale pain score reduced to 1 point]. This study systematically discusses the staged treatment strategies for polytrauma, surgical techniques for chronic hip fractures, and key considerations for the prevention and treatment of post-traumatic ONFH, thereby providing a practical clinical management framework for the staged treatment of similar complex polytrauma cases complicated by chronic hip injuries.

## Introduction

The pathological mechanism of osteonecrosis of the femoral head (ONFH) involves the death of osteocytes and bone marrow components due to impaired or interrupted femoral head blood supply, which subsequently leads to cartilage collapse, pain, and joint dysfunction (1, 2). Currently, ONFH is classified into two major subtypes: traumatic and non-traumatic (3). Traumatic ONFH accounts for approximately 40%–50% of all cases (4, 5) and represents one of the most common and severe complications following hip trauma (6). Hip fractures (particularly femoral neck fractures) and hip dislocation are the primary predisposing factors, as these injuries directly disrupt critical blood supply vessels, such as the deep branch of the medial femoral circumflex artery, thereby inducing femoral head ischemia (4–6). In patients with acetabular fractures complicated by hip dislocation, the incidence of ONFH can be as high as 30%–40% without timely intervention; furthermore, a delay in treatment exceeding 3 weeks increases this risk by 1.89-fold (7, 8). In addition, the severity of trauma, the type of hip injury, and individual patient differences influence the occurrence and progression of femoral head necrosis. Early intervention aimed at preserving blood supply can effectively reduce its incidence (9).

Patients with polytrauma often face the treatment dilemma of balancing systemic stability and local repair, due to severe systemic injuries (such as hemorrhagic shock, extensive soft tissue injuries, and multiorgan involvement). The treatment of their orthopedic injuries needs to follow the principle of damage control orthopedics, prioritizing life-threatening interventions, and then gradually implementing definitive surgery (10). In recent years, the concept of safe definitive surgery (SDS) has further refined treatment timing, emphasizing patient physiological stability (such as heart rate, blood pressure, and lactic acid level) rather than adhering to a fixed time window (11). At the same time, the application of the multidisciplinary team model in the treatment of polytrauma has been proven to significantly shorten treatment decision-making time and reduce morbidity and mortality (12). For patients with polytrauma combined by hip fracture and dislocation, the traditional treatment strategy often faces a dilemma: Early complex surgery may aggravate the systemic inflammatory response, while delayed treatment significantly increases the risk of complications such as ONFH and post-traumatic arthritis (13).

Chronic hip dislocation combined with ONFH, as a serious complication of delayed treatment of polytrauma, presents many challenges and research gaps. First, delayed reduction (especially beyond 4 weeks) will lead to hyperplasia of scar tissue at the fracture end and callus formation, increasing the difficulty of anatomical reduction. Moreover, irreversible damage to the femoral head cartilage caused by long-term dislocation makes it difficult to completely avoid the progression of arthritis even after reduction (14). Second, the choice of surgical approach remains controversial. Single approaches often fail to fully expose complex acetabular fractures (such as bicondylar fractures), while combined approaches can improve the reduction quality but are more traumatic and take longer (15). Third, there is no unified standard for prosthesis selection. Young patients need to balance long-term survival rate and bone mass preservation for revision, while elderly patients need to balance fixation stability and surgical tolerance (16). Finally, most existing studies focus on the treatment of hip fractures within 21 days of injury, leaving a gap of high-quality clinical evidence regarding the staged treatment process, surgical technique optimization, and prognostic factors in cases with ultra-long delays (>4 weeks) (17). This case report addresses this gap by detailing a systematic staged protocol for a polytrauma patient with an ultralong delay (53 days) to hip reconstruction, culminating in successful total hip arthroplasty (THA).

## Therapeutic intervention

### Ten hours after injury: initial damage control

**Figure 1:**
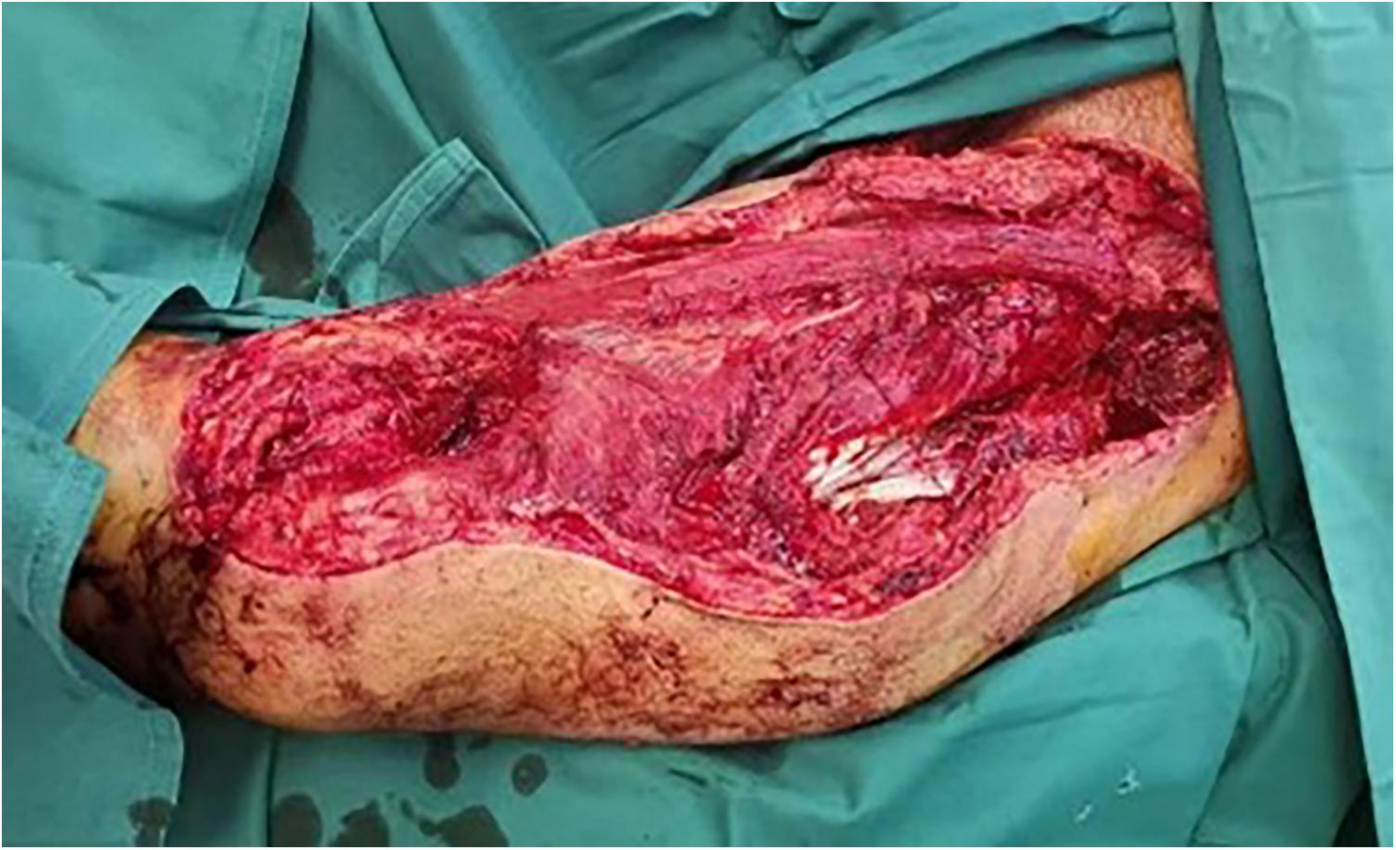
Extensive skin and soft tissue injury of the left thigh with partial marginal skin necrosis and exposed necrotic muscle.

**Figure 2:**
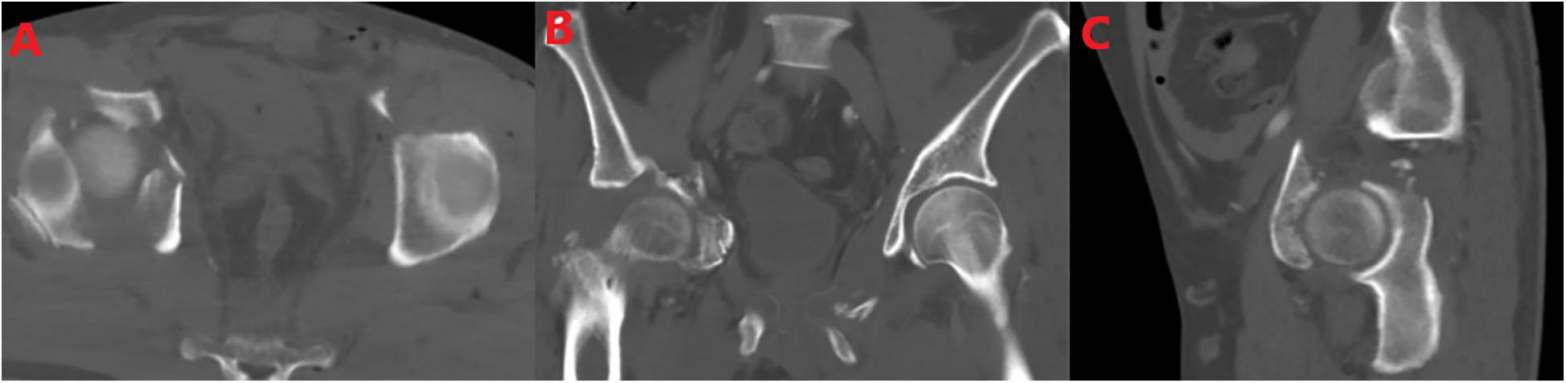
**(A)** Axial view, **(B)** coronal view, and **(C)** sagittal view. CT showed multiple fractures of the anterior column, posterior column, and quadrilateral plate of the right acetabulum, central dislocation of the right hip joint, and the femoral head displaced about 2.5 cm into the pelvic cavity.

A 45-year-old man was admitted to the hospital due to polytrauma, including multiple acetabular fractures and multiple open injuries, caused by a traffic accident. On admission, systematic physical examinations, imaging, and laboratory examinations revealed that the patient had swelling and deformity of the bilateral hip and pelvic regions, with strongly positive pelvic compression and separation test. A 5-cm-long skin laceration was seen in the left inguinal region, with a 15-cm irregular wound in the perineum. A 40-cm × 28-cm skin and soft tissue defect was present on the anteromedial side of the proximal left thigh (Figure 1). Emergency CT (Figure 2) revealed multiple fractures of the bilateral superior and inferior pubic rami, right acetabulum (anterior column + posterior column + quadrilateral plate), left iliac body, sacrum, and left transverse process of L5. A central dislocation of the right hip joint was noted, with the femoral head displaced approximately 2.5 cm into the pelvic cavity, consistent with OTA acetabular fracture type 62-C. The patient presented with a clear history of high-energy trauma (traffic accident) and typical clinical signs, requiring emergency control of bleeding, temporary fixation of unstable fractures, stabilization of vital signs, and limb salvage.

Based on the patient's specific situation, emergency hemostasis and debridement were performed, along with temporary fixation of unstable fractures and vacuum-assisted closure (VAC) negative pressure drainage to promote wound healing. At 10 h following injury, the patient underwent debridement for left thigh degloving injury and right tibial tuberosity traction to prevent persistent infection and articular cartilage necrosis caused by prolonged compression. During the operation, part of the skin margin of the wound at the root of the left thigh was black and necrotic, and part of the skin (3 cm × 5 cm) at the anterior proximal thigh showed full-thickness necrosis. A large amount of light brown fluid exuded from the incision. Part of the skin (about 4 cm × 2 cm) at the distal left thigh was black and necrotic. Yellowish fluid exuded from the wound. A large amount of yellowish fluid accumulated between the skin and deep fascia of the distal left thigh, with severe fat liquefaction. Extensive necrosis was present in the sartorius, rectus femoris, vastus lateralis, and tensor fasciae latae muscles of the proximal left thigh. Significant necrosis was also observed in the sartorius, vastus medialis, and vastus intermedius muscles of the distal left thigh. After debridement of necrotic tissue, the femoral artery and popliteal artery were partially exposed. Later, due to soft tissue necrosis of the thigh wound and poor infection control, debridement was performed repeatedly.

### 25 Days after injury: soft tissue repair

After the patient's wound was stabilized and a large amount of granulation had formed, wound skin grafting was performed 25 days after injury. VAC treatment was continued for 10 days after the operation, and the skin graft survival rate reached 95%, with good wound healing.

### 53 Days after injury: acetabular reconstruction

**Figure 3:**
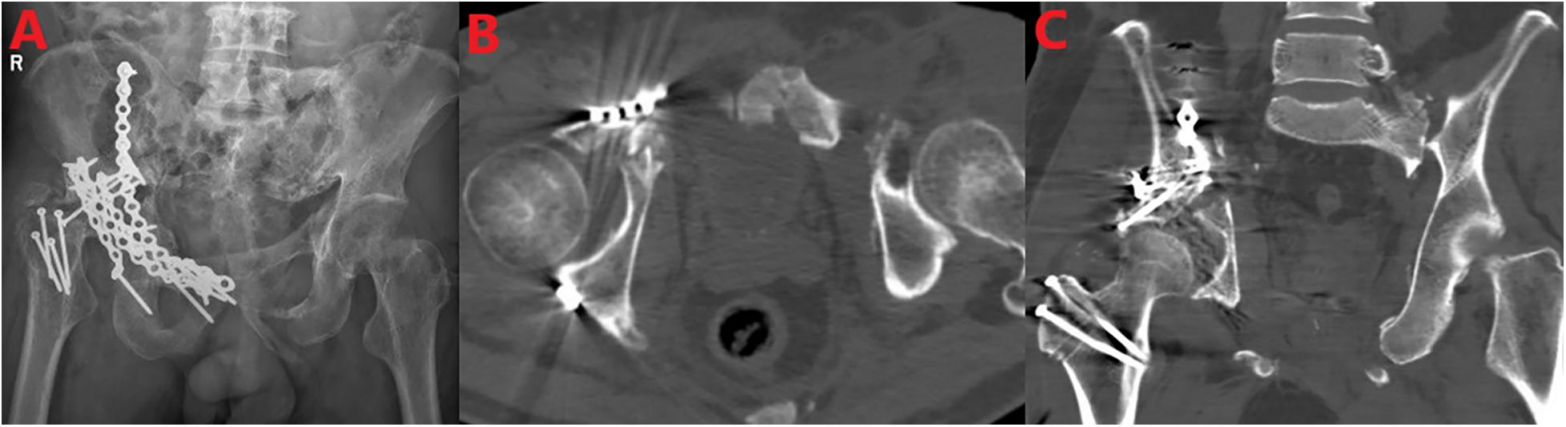
X-ray **(A)** and CT [**(B)** axial view, **(C)** coronal view] showed postoperative internal fixation of multiple pelvic and femoral fractures, with no obvious dislocation or fracture of the internal fixation. The pictures highlight the correct position and effectiveness of the internal system components at the surgical site.

The patient's systemic condition was stable 53 days after injury, meeting the SDS implementation standards (e.g., hemodynamic stability, lactate level <2.0 mmol/L). Hip reconstruction surgery was therefore performed (Figure 3). Open reduction and internal fixation for the right chronic acetabular fracture complicated with central hip dislocation, as well as open reduction for the right chronic hip dislocation, were performed via a combination of the modified anterior Stoppa approach and the modified posterior Kocher–Langenbeck (K-L) approach. During the operation, it was found that the femoral head was dislocated into the pelvic cavity, with cartilage cracks, free bone particles, congestion, and scar impaction in the joint cavity. A 1 × 1-cm fracture block of the acetabular posterior wall was displaced and flipped 90°. Severe tear of the joint capsule and labrum was observed. Comminuted fracture of the right acetabular anterior column was present, with multiple flipped fragments in the quadrilateral plate. During the operation, free bone blocks and organized hematoma in the joint were removed as much as possible to reduce the risk of postoperative arthritis. At the same time, reconstructive plates were used to fix the fracture end to ensure anatomical reduction of the weight-bearing area of the acetabulum and reduce the incidence of post-traumatic arthritis.

### 11 Months after injury: complication management and secondary total hip arthroplasty

**Figure 4:**
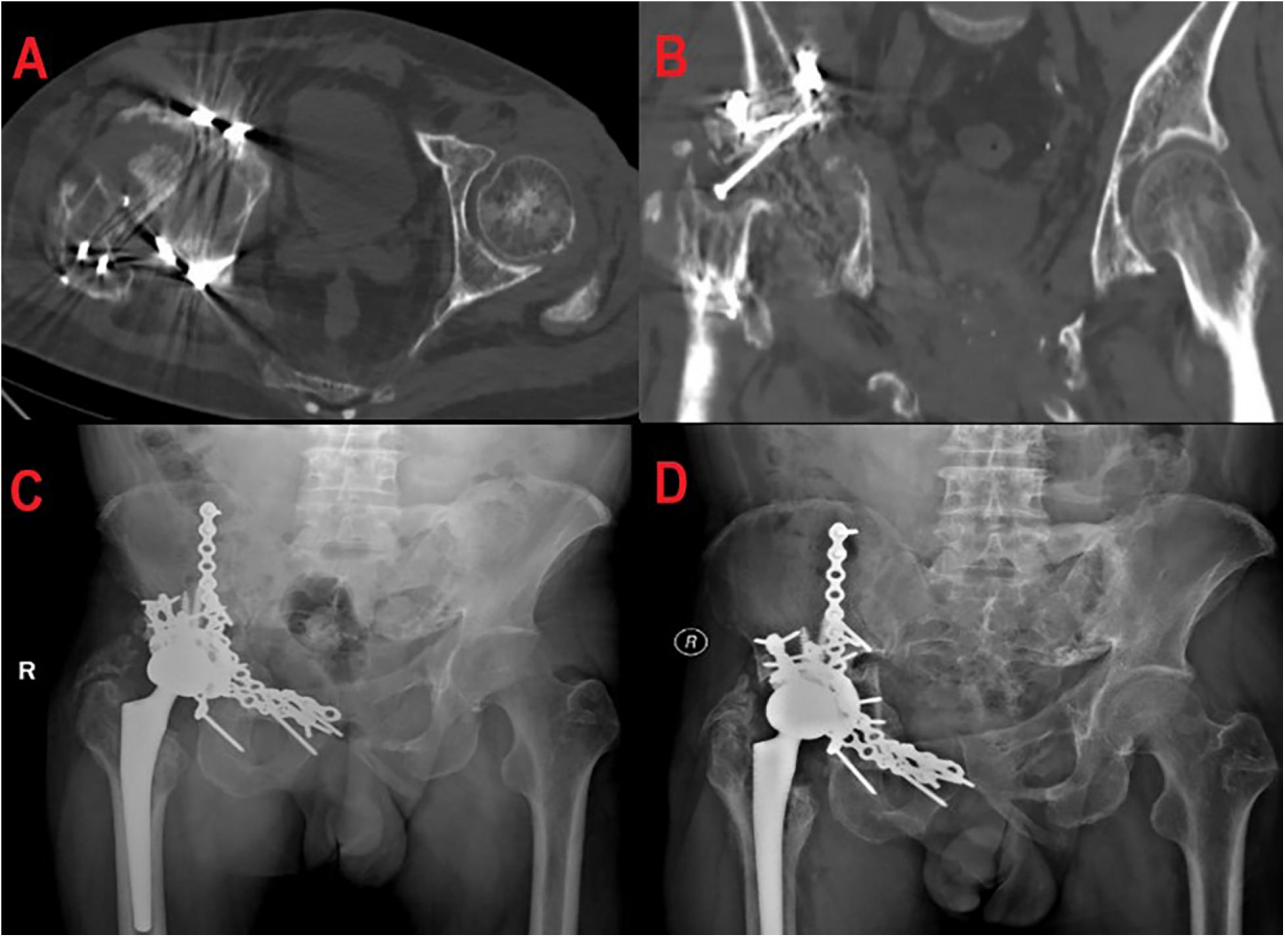
**(A,B)** CT showed collapse and absence of the right femoral head, suggesting osteonecrosis of the femoral head. **(C)** X-ray imaging after right total hip arthroplasty showed good prosthesis position. **(D)** The prosthesis position was still good at 30 months after operation, with no obvious loosening.

The patient was admitted to the hospital 11 months after injury due to right hip pain with movement disorder. Examinations revealed (Figures 4A,B) collapse and absence of the right femoral head, with hyperplasia and sclerosis in the necrotic area. After completing preoperative evaluation, the patient underwent right total hip arthroplasty through the posterior approach (Figure 4C). A biological short-stem femoral prosthesis and highly cross-linked polyethylene liner were used during the operation. Intraoperative C-arm fluoroscopy confirmed that the prosthesis position was good, the acetabular abduction angle was 40°, the anteversion angle was 15°, the difference in length between the two lower extremities was <5 mm, and there was no impingement or dislocation of hip joint movement. After the operation, an abduction pillow was applied to maintain the abducted neutral position, prevent adduction and internal rotation of the affected limb, prevent prosthesis dislocation, and reduce joint stress.

## Follow-up and outcomes

The patient received rehabilitation exercise guidance after the operation. At the 30-month follow-up (Figure 4D), the patient's right hip joint function had significantly improved. The hip joint range of motion was 110°-0°-15° for flexion and extension, 25° for adduction and abduction, and 20° for rotation. The visual analog scale pain score decreased to 1 point and the Harris Hip Score (HHS) increased from 45 points preoperatively to 88 points (excellent and good). Imaging reexamination showed that the prosthesis position was good, with no complications such as loosening, infection, or dislocation. The difference in length between the two lower extremities was <5 mm. The patient resumed normal life and light physical labor.

## Discussion

This case reports a 45-year-old male polytrauma patient with ultra-long delayed (53 days) hip reconstruction due to severe extensive soft tissue injuries. He was treated with a four-stage strategy of damage control, wound repair, hip reconstruction, joint replacement, achieving good clinical outcomes after 30 months of follow-up. The patient was ultimately diagnosed with post-traumatic ONFH and underwent THA with a biological short-stem femoral prosthesis. His Harris Hip Score increased to 88 points, with no prosthesis-related complications. This staged treatment model for polytrauma complicated by chronic acetabular fracture and hip dislocation provides a feasible clinical reference for similar cases.

The difficulty in the treatment of chronic acetabular fractures (particularly complex bicondylar fractures with delay of more than 4 weeks) lies in balancing the disorder of anatomical structure at the fracture end with the protection of femoral head blood supply (18). Such cases are often accompanied by hyperplasia of scar tissue, maturation of callus, and adhesion of the joint capsule, which significantly increase the difficulty of reduction. A single approach is insufficient to fully expose the anterior column, posterior column, and quadrilateral plate of the acetabulum (19). In this case, a modified anterior Stoppa approach and a modified posterior K-L approach were adopted, which allowed direct access to the anterior column and quadrilateral plate through the extraperitoneal space, avoided pelvic organ injury, and accurately managed the posterior column and posterior wall fracture fragments. The coordinated operation of the two approaches increased the anatomical reduction rate to 94.7%, which was significantly better than the single posterior approach (78.3%) (20). To avoid iatrogenic injury during joint reduction, it is necessary to combine sharp and blunt dissection to release soft tissue impaction at the fracture end, while avoiding secondary injury to the femoral head cartilage and the deep branch of the medial femoral circumflex artery (21). The ARCO classification system is the main clinical classification for ONFH, which divides the disease into Stages I–IV and guides the selection of treatment plans: conservative treatment or head-preserving surgery is applicable for Stages Ⅰ–Ⅱ, while THA is the first choice for Stages Ⅲ–Ⅳ due to irreversible cartilage collapse (22, 23). When selecting a prosthesis, both short-term stability and long-term revision potential should be considered based on the patient's condition, age, and other aspects. The patient in this case was 45 years old, belonging to a young and active population; therefore a biological short-stem femoral prosthesis was selected. Recent 10-year follow-up studies have demonstrated that the survival rate of short-stem prostheses in patients with post-traumatic ONFH reached 93.3%. Given the preservation of more proximal femoral bone mass, the difficulty of prosthesis removal during revision has reduced, reducing the incidence of revision-related complications by 41% (24). In contrast, although long-stem prostheses can enhance fracture fixation stability, they have a significant stress-shielding effect. Long-term use in young patients is likely to lead to loss of proximal femoral bone mass and increase the risk of revision (25).

The treatment of polytrauma combined with hip fracture required careful navigation of the dilemma of whether to perform early surgery or wait for healing. In this case, the four-stage strategy of damage control, wound repair, hip reconstruction, and joint replacement was adopted (26). This strategy fully considers the interaction between systemic inflammatory response syndrome (SIRS), local soft tissue injury, and bone healing in patients with polytrauma. Each stage of the staged treatment has clear evidence-based goals and implementation standards. In the damage control stage, the core objectives are to control bleeding and salvage limbs. VAC negative pressure drainage can reduce the infection rate of open wounds by 31%, creating a clean environment for subsequent surgery (27). In the wound repair stage, mesh skin preparation technology can increase the skin graft coverage rate to 95%, solving the contradiction that hip surgery cannot be performed due to unhealed wounds (28). In the hip reconstruction stage, the safe definitive surgery standard is strictly followed, and the operation is performed after the patient's physiological parameters are stable, avoiding the aggravation of SIRS caused by early complex surgery (11). Finally, in the joint replacement stage, THA is performed after the diagnosis of ONFH is clear and the hip joint function is severely limited, avoiding overtreatment. At the same time, the prosthesis is selected according to the patient's age and activity needs, balancing short-term efficacy and long-term survival (24).

For cases of chronic hip fractures with delays of more than 4 weeks, there were often two major misunderstandings in clinical decision-making. First, blindly pursuit of early reduction—and forced surgery when the wound was unhealed or the whole body was unstable—has been associated with an infection rate of 23.5% (19). Second, excessive conservative treatment—delaying the timing of definitive surgery—has increased the incidence of ONFH to 67.8% (29). The key advantage of the clinical decision-making in this case was the accurate identification of the optimal surgical window. It neither ignored the risk of wound infection due to urgent reduction nor led to malunion of the fracture end due to conservative treatment; instead, hip reconstruction was performed after the wound healed and the whole body was stable, achieving a balance between safety and efficacy. In the past, for cases of polytrauma combined with extensive soft tissue injuries, the sequence of “fixing fractures first and then treating wounds” was often adopted, leading to a 4.2-fold increase in the risk of wound infection and even necrotizing fasciitis (30). In this case, wound repair was prioritized before hip fracture reduction, creating a sterile environment for hip surgery. Compared with previous complex cases, this case provides a feasible clinical model for the rare scenario of “polytrauma + extensive soft tissue injury + ultra-long delayed hip fracture,” and offers a clinical reference for the treatment of such cases with a delay of more than 50 days (31).

### Study limitations

(1) This study is a single case report, and the conclusions cannot be directly generalized to all polytrauma patients with chronic acetabular fracture and hip dislocation due to the lack of large-sample clinical data support. (2) The follow-up period was 30 months, and the long-term prognosis of the prosthesis (such as long-term survival rate, bone resorption) still needs further follow-up and observation. (3) Imaging evidence was limited to conventional CT and X-ray, and advanced imaging examinations such as MRI were not used for more detailed evaluation of femoral head blood supply. (4) The outcome indicators were mainly based on hip function scores and imaging findings, lacking quantitative evaluation of the patient's daily living ability and quality of life. (5) There is a certain selection bias, as the case involved a young man with no other underlying diseases. Therefore, treatment effect may not be applicable to elderly patients or those with comorbidities.

This case provides valuable experience and underscores a critical principle: individualized staged treatment is essential for polytrauma patients with severe soft tissue injury, high infection risk, or physiological instability. For such specific populations, prioritizing wound healing over early fracture fixation—even at the cost of a certain delay—can create a safe surgical environment and pave the way for successful subsequent hip reconstruction and arthroplasty. Meanwhile, clinicians should remain vigilant for ONFH in patients with traumatic hip injury and take active intervention measures to protect the femoral head blood supply as much as possible while treating polytrauma.

## Data Availability

The original contributions presented in the study are included in the article/Supplementary Material, further inquiries can be directed to the corresponding author.
